# Pan-cancer analysis of co-occurring mutations in RAD52 and the BRCA1-BRCA2-PALB2 axis in human cancers

**DOI:** 10.1371/journal.pone.0273736

**Published:** 2022-09-15

**Authors:** Abdulaziz B. Hamid, Lauren E. Frank, Renee A. Bouley, Ruben C. Petreaca

**Affiliations:** 1 Medical College of Wisconsin, Milwaukee, WI, United States of America; 2 Zoology Undergraduate Program, The Ohio State University, Marion, OH, United States of America; 3 Department of Chemistry and Biochemistry, The Ohio State University, Marion, OH, United States of America; 4 Department of Molecular Genetics, The Ohio State University at Marion, Marion, OH, United States of America; 5 Cancer Biology Program, The Ohio State University James Comprehensive Cancer Center, Columbus, OH, United States of America; Tulane University Health Sciences Center, UNITED STATES

## Abstract

In human cells homologous recombination (HR) is critical for repair of DNA double strand breaks (DSBs) and rescue of stalled or collapsed replication forks. HR is facilitated by RAD51 which is loaded onto DNA by either BRCA2-BRCA1-PALB2 or RAD52. In human culture cells, double-knockdowns of RAD52 and genes in the BRCA1-BRCA2-PALB2 axis are lethal. Mutations in BRCA2, BRCA1 or PALB2 significantly impairs error free HR as RAD51 loading relies on RAD52 which is not as proficient as BRCA2-BRCA1-PALB2. RAD52 also facilitates Single Strand Annealing (SSA) that produces intra-chromosomal deletions. Some RAD52 mutations that affect the SSA function or decrease RAD52 association with DNA can suppress certain BRCA2 associated phenotypes in breast cancers. In this report we did a pan-cancer analysis using data reported on the Catalogue of Somatic Mutations in Cancers (COSMIC) to identify double mutants between RAD52 and BRCA1, BRCA2 or PALB2 that occur in cancer cells. We find that co-occurring mutations are likely in certain cancer tissues but not others. However, all mutations occur in a heterozygous state. Further, using computational and machine learning tools we identified only a handful of pathogenic or driver mutations predicted to significantly affect the function of the proteins. This supports previous findings that co-inactivation of RAD52 with any members of the BRCA2-BRCA1-PALB2 axis is lethal. Molecular modeling also revealed that pathogenic RAD52 mutations co-occurring with mutations in BRCA2-BRCA1-PALB2 axis are either expected to attenuate its SSA function or its interaction with DNA. This study extends previous breast cancer findings to other cancer types and shows that co-occurring mutations likely destabilize HR by similar mechanisms as in breast cancers.

## Introduction

Homologous recombination (HR) is a crucial process for repair of DNA double strand breaks that may arise from exogenous insults or because of endogenous processes such as stalled or collapsed replication forks [1–4]. HR evolved first in bacteria to facilitate accurate replication of long genomes which are prone to chromosome breaks [5]. In eukaryotes, the indispensable HR process was retained and was also subsequently adapted for meiosis. A clue to the importance of HR in DSB repair is the conservation of both structure and function of the genes involved [6, 7].

HR is mediated by members of the RAD52 epistatic group which are conserved from yeast to humans [7]. Of these, RAD51 is central to recombination. It nucleates the resected single stranded DNA and prepares the broken ends for homology search and strand invasion [8, 9]. The gene is essential in humans and compounds have already been developed to inhibit its function in cancer cells to sensitize them to DNA damaging therapeutic agents [10].

RAD51 mediated HR generally results in error-free repair but requires the breast cancer susceptibility gene BRCA2 which facilitates RAD51 loading onto the resected single stranded DNA [11]. In human cells, RAD52 can substitute for the BRCA2 function in loading RAD51 [12–14]. Yeast does not have BRCA2 and RAD52 is singularly responsible for loading RAD51 [15–18]. However, in human cells the function of RAD52 has been historically largely ignored but more recent evidence shows that it plays an important role in HR [19] particularly in cells with BRCA2 mutations [20–22]. Additionally, RAD52 is required for mediating the RAD51-independent backup Single Strand Annealing (SSA) pathway which functions primarily at regions of direct repeats [19, 23–28]. The SSA pathway produces intra-chromosomal deletions and is mutagenic [28].

In line with the observation that RAD52 and BRCA2 may independently facilitate RAD51 function, it was shown that knockdown of both RAD52 and BRCA2 in cell cultures is lethal [29]. RAD51 foci were significantly decreased in the double knockdown cells. Furthermore, inhibiting RAD52 in cancer cells harboring BRCA2 mutations can selectively and effectively kill cells [30–32]. Conversely, over-expressing RAD52 can rescue some phenotypes associated with BRCA2 loss [20]. A RAD52 S346X truncation variant with reduced SSA activity decreases the risk of developing breast cancer in women with BRCA2 germline mutations [21, 22]. However, the N-terminus domain of RAD52 is required to sustain repair in cells with deficient BRCA2 activity [33]. This observation suggests that although RAD52 can substitute for BRCA2, its SSA activity is mutagenic. Additionally, the essential function of RAD52 resides in the N-terminus.

Synthetic lethality accompanied by decrease in RAD51 foci was also observed in cells depleted of RAD52 and the other breast cancer susceptibility gene, BRCA1 [34]. BRCA1 has a more pleiotropic role than BRCA2. In addition to facilitating the BRCA2/RAD51 function it also participates in DNA end resection, DNA damage checkpoint activation and recruitment of chromatin remodeling complexes [35]. Finally, RAD52 is also lethal with PALB2 [34], a factor that interacts with both BRCA1 and BRCA2 and facilitate their function [36]. All three genes (BRCA1, BRCA2, PALB2) are required for efficient HR repair. These experiments suggest that RAD52 works in parallel with BRCA1-BRCA2-PALB2 to modulate the function of RAD51.

A functional BRCA1/BRCA2/PALB2 module is required for error-free repair [37–39]. The highest level of chromosomal aberrations is observed in cells that harbor both BRCA2 and RAD52 mutations compared with single mutants [29] and RAD52 is required for HR when BRCA1 or PALB2 are mutated [34]. Even in yeast, proper loading of RAD51 is required for suppressing chromosomal re-arrangements particularly at regions of tandem repeats [40].

Public cancer genomes databases make it possible to screen data for mutation signatures. The Catalogue of Somatic Mutations in Cancers (COSMIC) [41] deposits cancer genomes data from various sources including the NIH TCGA project. Here we queried this database for co-occurring RAD52-BRCA2, RAD52-BRCA1 and RAD52-PALB2 mutations. We find pathogenic or driver co-occurring mutations in these gene pairs albeit always in heterozygous status. We describe and characterize these mutations.

## Materials and methods

Mutation data for RAD52, BRCA1, BRCA2, and PALB2 were downloaded as excel files (.csv) from the COSMIC database (https://cancer.sanger.ac.uk/cosmic accessed in May 2021, version 94, hg38). COSMIC provides identifiers for every mutation (SAMPLE_NAME). Using these identifiers, we compared the four files and extracted co-occurring mutations.

To identify pathogenic or driver mutations we used OPEN-CRAVAT (Cancer-Related Analysis of Variants Toolkit, https://opencravat.org) [42, 43]. Within this platform we used the CHASMPlus tool which characterizes mutations as either silent or driver [44] and VEST4 [45, 46] which calculates the probability of mutations being pathogenic.

The cBioPortal (www.cbioportal.org) interphase was used extract statistical values for co-occurrence or exclusivity [47, 48]. For the data in **Fig 2C** we chose “TCGA PanCancer Atlas Studies” while for the data in **Fig 2D** and **S2 Table** we computed values for individual cancers by selecting them from the tissues on the left side of the website. For the individual cancers overlapping samples were excluded. All three parameters (mutations, structural variants, and copy number alterations) were chosen. The statistical values were extracted using the “Mutual exclusivity” tab. Both p-values and q-values were extracted for all gene combinations, but the Log2 odds ratio is a prognosticator of co-occurrence or exclusivity. A Log2 ratio above 0 indicates co-occurrence while a ratio below 0 indicates exclusivity.

SWISS-MODEL [49] was used to generate homology models of the point mutants. PyMOL Molecular Graphics System version 2.3.4 was used to align structures and prepare figures.

Lollipops representing mutation distribution were made using the MutationMapper tool of cBioPortal (https://www.cbioportal.org) [47, 48].

Data was analyzed with SPSS and all figures were prepared using Photoshop.

## Results and discussion

### Distribution of RAD52, BRCA2, BRCA1 and PALB2 mutations

**Fig 1:**
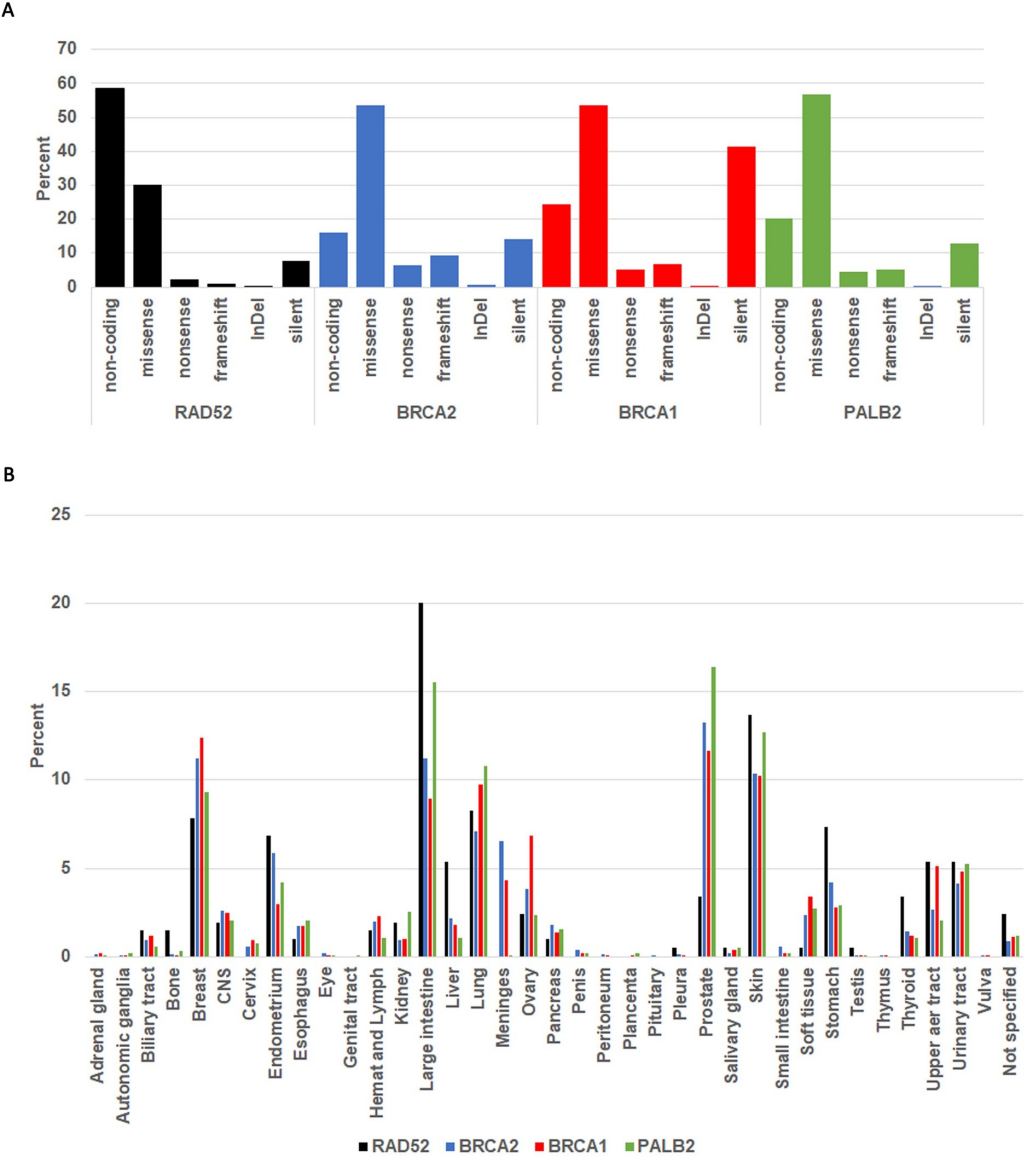
Distribution of RAD52, BRCA2, BRCA1 and PALB2 mutations in cancer cells. **A**. Distribution by type of mutation. The COSMIC mutations were partitioned into non-coding (5’ UTR, 3’UTR and intronic) and coding (translated). The coding mutations were further partitioned into missense, non-sense, frameshift, InDels and silent. All frameshift mutations introduce a stop codon and are therefore truncations. **B**. The distribution of all coding RAD52, BRCA2, BRCA1 and PALB2 among the various cancers reported on COSMIC. The large intestine, breast, prostate and skin are the most represented cancers.

We partitioned the mutations reported on COSMIC into non-coding (5’UTR, 3’UTR and intronic), and coding (within translated sequence). The coding mutations were further partitioned into missense, nonsense, frameshift, InDels (small insertions and deletions) and silent (**Fig 1A**). Mutations in every category are found in all four genes. Missense mutations are the most abundant in BRCA1 (53.6%), BRCA2 (53.6%) and PALB2 (56.8%) but not RAD52 (30.2%). Most mutations in RAD52 are non-coding (58.4%). The significance of this observation is not immediately clear. It is not explained by the percent of the coding sequence (percent exons) which is 8.3% RAD52, 14.2% BRCA2, 8.7% BRCA1 and 10.7% PALB2. BRCA1 also has a high percentage of silent mutations (41.5%). Silent mutations can change codon optimization and affect translation efficiency [50–52]. We next mapped the distribution of coding mutations in the reported cancer primary sites (**Fig 1B**). Most mutations reported on COSMIC occur in the breast, large intestine, lung, prostate and skin cancers. Mutations in all four genes are found in all tissues generally equally except for the large intestine and prostate. Remarkably, large intestine cancers are characterized by a higher ratio of inter-chromosomal to intra-chromosomal aberrations while the prostate has a higher ratio of intra-chromosomal to inter-chromosomal aberrations [53]. This observation may reflect the role of RAD52 in promoting more intra-chromosomal repair (e.g., SSA) while BRCA1-BRCA2-PALB2 may facilitate inter-chromosomal aberrations. However, this analysis is speculative since most repair in human cells occurs by non-HR pathways (e.g., Non_Homologous_End_Joining (NHEJ) and related pathways) [54–56].

### Co-occurring mutations in cancer tissues

**Fig 2:**
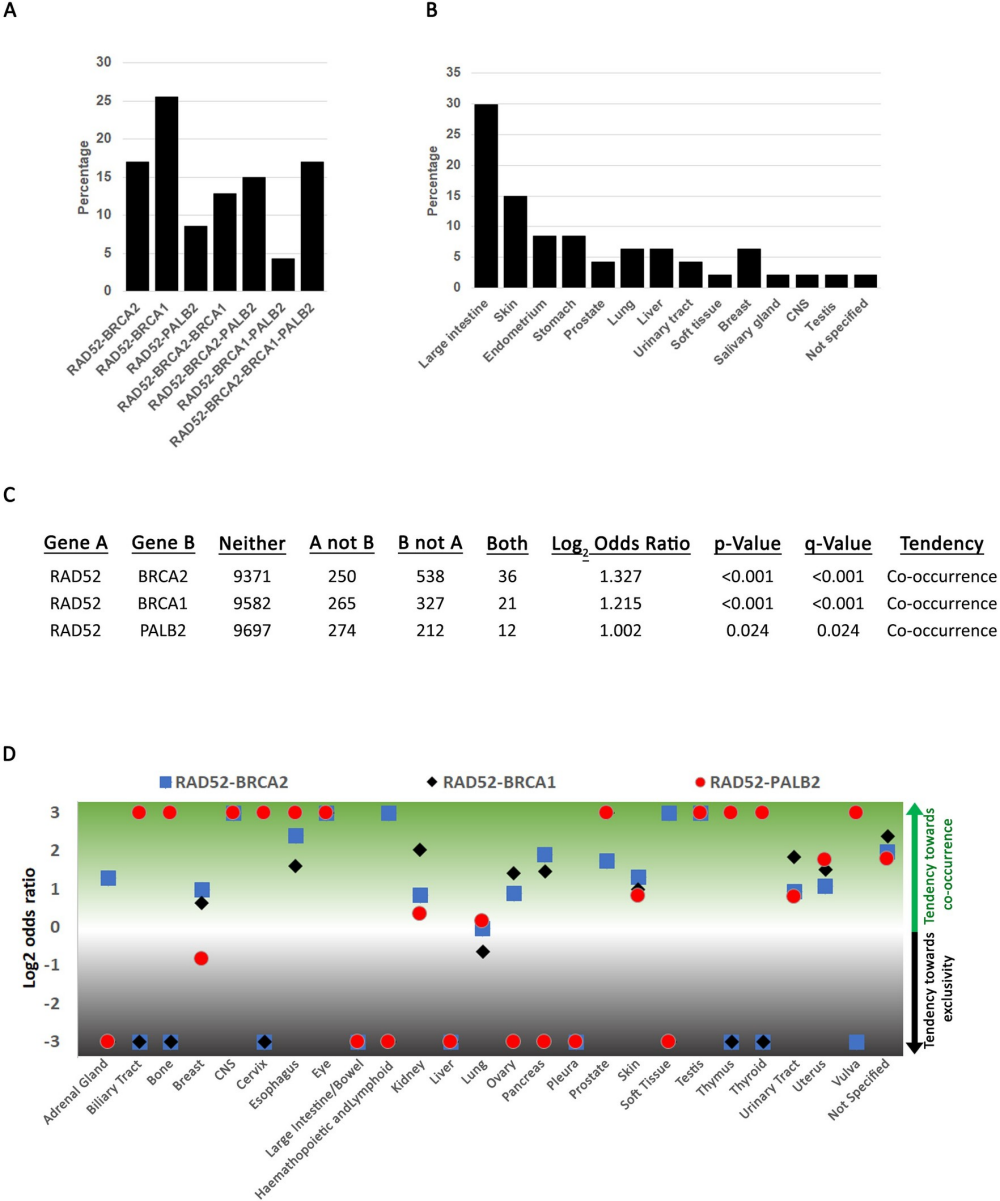
Co-occurring mutations in RAD52 and the BRCA2-BRCA1-PALB2 axis. **A**. Percent co-occurring mutations between the four genes using data from **Table 1**. Total percentage (100%) represents all mutations in **Table 1**. **B**. Distribution of co-occurring mutations among the different cancer types. A graphical representation of the data from **Table 1**. **C**. Pan-cancer statistical analysis if co-occurring mutations using cBioPortal (www.cbioportal.org). **D**. Statistical analysis of co-occurring mutations by tissue using cBioPortal.

We next searched for RAD52 co-occurring mutations with BRCA2, BRCA1 and PALB2. This analysis was possible because COSMIC reports a sample identifier (SAMPLE_NAME) and we searched for commutated genes in each sample. Of the cases that reported one coding RAD52 mutation, we identified 22% that also had a mutation in either BRCA2, BRCA1, or PALB2 (**S1 Table**, **Fig 2A**). Some mutations occurred in more than two genes (**Fig 2A**). Most co-occurring mutations were identified in the large intestine and skin (**Fig 2B**). This may suggest that some tissues do not tolerate double mutants in both RAD52 and the BRCA1-BRCA2-PALB2 axis. Mutation zygosity is reported for some but not all mutations and when reported, all co-occurring mutations are heterozygous. Co-occurring mutations were identified in all regions of the four genes; there was no cold region (**S1 Fig**).

To understand whether co-occurring mutations are statistically significant, we used the cBioPortal (www.cbioportal.org), a web interphase for statistical analysis [47, 48]. The algorithm can predict whether mutations in any two gene pairs are likely to co-occur or display mutual exclusivity (meaning that it is unlikely to find statistically significant co-occurring mutations). We first carried out a pan-cancer analysis using the TCGA Pan-Cancer Atlas Studies (**Fig 2C**). Remarkably, we find that there is a tendency of co-occurring mutations between RAD52 and BRCA2, BRCA1 or PALB2. We next checked for co-occurring mutations by cancer type (**Fig 2D** and **S2 Table**). We find that each cancer is distinct in its tendency of co-occurrence or exclusivity. In breast cancers, esophagus, kidney, lung, prostate, skin, testis, urinary tract, and uterus there is a tendency towards co-occurring mutations between all gene pairs. In certain tissues such as the CNS, eye, prostate and testis, the tendency towards co-occurring mutations between any gene pair is high. Remarkably, several tissues show mutual exclusivity primarily between RAD52 and PALB2 or RAD52 and BRCA1 (adrenal gland, large intestine, hematopoietic and lymphoid, liver, ovary, pancreas, pleura, and soft tissue).

Only the large intestine shows mutual exclusivity between any pair combination. A recent pan-cancer reports that colorectal cancers are characterized by fragile sites prone to deletions or duplications [57]. However, a comprehensive genetic analysis of colorectal cancers found that neither RAD52 nor BRCA1, BRCA2 or PALB2 are significantly mutated but the NHEJ pathway was significantly altered [58]. Chromosomal instability in colorectal cancers is associated with aggressiveness and poor survival [59] but there is high concordance between mutations in primary vs metastatic colorectal cancers [60]. Thus, it appears that NHEJ may be the major driver of instability in colorectal cancers.

### Mutation impact on gene function

**Fig 3:**
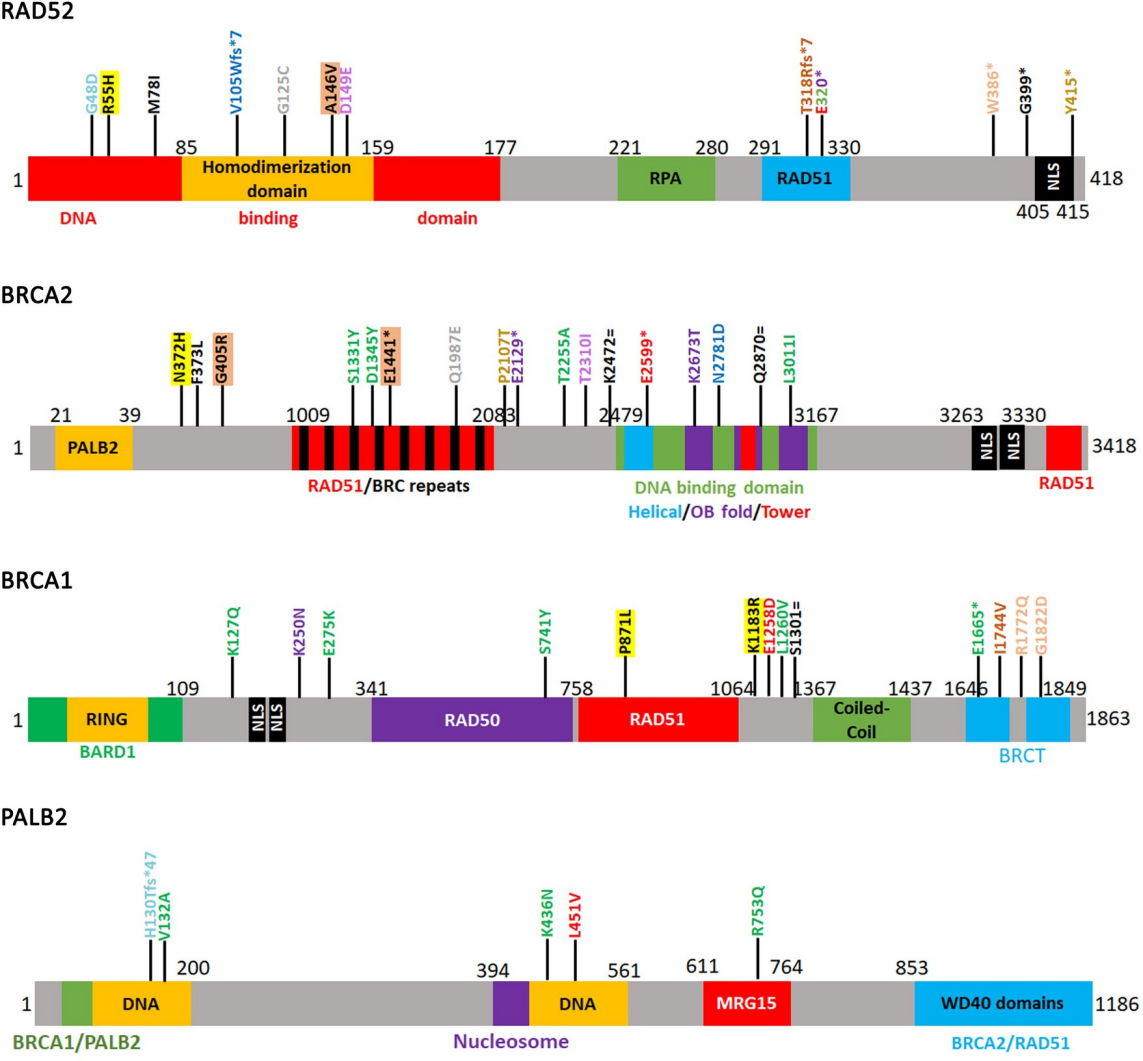
Significant mutations in RAD52, BRCA2, BRCA1 and PALB2. Cartoon diagrams of RAD52, BRCA2, BRCA1 and PALB2 showing specific domains in each gene. The diagrams were adapted from [19, 61–64]. Co-occurring mutations from **Table 1** are color coded. Highlighted mutations are those with a high probability of being driver or pathogenic.

Not all mutations impact gene function equally. To determine which mutations are likely to have a significant impact on gene function, we employed two machine learning or computational tests: the Cancer Specific High Throughput Annotation of Somatic Mutations (CHASM) and the Variant Effect Scoring Tool (VEST) (**S3A–S3D Table**). CHASM classifies mutation as either driver or passengers [44] while VEST is a machine learning algorithm that predicts the probability of a pathogenic mutation [45]. The algorithms generate a p-value. Those mutations with a p-value of 0.05 or below are interpreted as either driver (CHASM) or pathogenic (VEST). We then mapped all mutations on cartoon diagrams of RAD52, BRCA2, BRCA1 and PALB2 adapted from [19, 61–64]. When we retrieved all mutations with a significant probability of being either driver or pathogenic, we identified only two instances where co-occurring mutations are likely to significantly alter the function of the genes (**Table 1, Fig 3**).

**Table 1:** Co-occurring mutations likely to be driver or pathogenic.

Tissue	RAD52			BRCA2			BRCA1			PALB2			RAD51		
	MUT	CHASM^1^	VEST^2^	MUT	CHASM	VEST	MUT	CHASM	VEST	MUT	CHASM	VEST	MUT	CHASM	VEST
Large intestine	***R55H*** ^***3***^	0.0989	0.0028	***N372H***	0.0427	0.4127	**P871L**	0.0002	0.3354	N/A	N/A	N/A	N/A	N/A	N/A
							***K1183R***	0.0053	0.6445						
Large intestine	G48D	0.0695	0.0052	N/A	N/A	N/A	N/A	N/A	N/A	H130Tfs*47	N/A	N/A	N/A	N/A	N/A
Skin	D149E	0.102	0.0067	T2310I	0.1	0.1534	N/A	N/A	N/A	N/A	N/A	N/A	N/A	N/A	N/A
Skin	G125C	0.083	0.0007	Q1987E	0.0549	0.4446	N/A	N/A	N/A	N/A	N/A	N/A	N/A	N/A	N/A
Endometrium	M78I	0.185	0.0062	F373L	0.0797	0.5986	Z = 2.08^4^	N/A	N/A	N/A	N/A	N/A	Z = 2.210	N/A	N/A
	G399*	N/A	0.2282	K2472 = Q2870 =	N/A	N/A									
					N/A	N/A									
Endometrium	***A146V***	0.0925	0.0035	E1441*	N/A	0.0217	Z = 2.587	N/A	N/A	N/A	N/A	N/A	N/A	N/A	N/A
				***G405R***	0.0520	0.0222									
**RAD52 truncating mutations**															
Endometrium	V105Wfs*7	N/A	N/A	N2781D	0.0154	0.0185	S1301 =	N/A	N/A	N/A	N/A	N/A	N/A	N/A	N/A
Urinary tract	T318Rfs*5	N/A	N/A	N/A	N/A	N/A	I1744V	0.0001	0.2157	N/A	N/A	N/A	N/A	N/A	N/A
^5^Large intestine	E320*	N/A	0.1396	E2599*	N/A	0.02384	E1258D	0.0017	0.2260	L451V	0.6910	0.9617	Z = 2.158	N/A	N/A
Large intestine	E320*	N/A	0.1396	E2129*	N/A	0.01929	K250N	0.0026	0.2003	N/A	N/A	N/A	N/A	N/A	N/A
				K2673T	0.0542	0.2121									
Large intestine	E320*	N/A	0.1396	S1331Y	0.0950	0.3885	E1665*	N/A	0.0179	K436N	0.5190	0.5288	N/A	N/A	N/A
				D1345Y	0.0649	0.2982	E275K	0.0041	0.2265	R753Q	0.7210	0.9130			
				T2255A	0.0505	0.3591	K127Q	0.0071	0.2565	V132A	0.6910	0.8089			
				L3011I	0.0295	0.3591	L1260V	0.0075	0.3166						
							S741Y	0.0022	0.1891						
Not specified	W386*	N/A	N/A	N/A	N/A	N/A	G1822D	0.0065	0.2592	N/A	N/A	N/A	N/A	N/A	N/A
							R1772Q	0.0007	0.0044						
Soft tissue	Y415*	N/A	0.2274	N372H	0.0427	0.4127	Z = 2.08	N/A	N/A	N/A	N/A	N/A	Z = 2.212	N/A	N/A
				P2107T	0.0686	0.4954									

^1^Shown are p-values computed by OpenCRAVAT using the CHASM algorithm. A p-value of 0.05 or below represents a statistically significant driver mutation.

^2^Shown are p-values computed by OpenCRAVAT using the VEST4 algorithm. A p-value of 0.05 or below represents a statistically significant pathogenic mutation.

^3^Co-occuring mutations are highlighted and represented graphically in Fig 3. More information on each mutation as well as a PubMed ID (if available) for the manuscript where the mutation was first reported is presented in S3E Table.

^4^The Z-score represents expression level for TCGA samples. A Z-score >2 is interpreted as over-expressed while a Z-score <2 is interpreted as under-expressed.

^5^This mutation appears in three different patients.

One sample was identified in the large intestine in a colorectal carcinoma with liver metastasis (**S3E Table**) [65]. The R55H mutation falls in the RAD52 DNA binding domain (**Fig 3**). The commutated BRCA2 N372H is situated in a region between the PALB2 and RAD51 binding domains. The BRCA1 P871L mutation is in the RAD51 binding domain while K1183R is situated right outside the binding domain. This sample was also characterized by hypermutation, increased copy number variation and a high frequency of LOH. Missense mutations in this sample were found in two DNA polymerase genes (POLQ, POLG) and the Bloom helicase (BLM) in addition to a variety of other genes. These findings are consistent with previous observations that metastatic colorectal cancers are characterized by increased chromosomal instability and are likely to have mutations in DNA damage repair genes [59].

We identified a second sample in the endometrium. This sample had a mutation in the RAD52 homodimerization domain (A146V) and two mutations in BRCA2: G405R situated between the PALB2 and RAD51 interaction domains and E1441* which truncates the C-terminus from RAD51 binding domain/BRC repeats (**Fig 3**). Additionally, BRCA1 was overexpressed (Z = 2.5872). This sample was characterized by a C/G>T/A mutational signature indicative of an endogenous mutation process known as “clocklike signature” [66]. Other identified mutations in the sample were in the protocadherin gamma subfamily genes which are often associated with advanced cancers and metastasis [67, 68]. Mutations were also identified in Deleted in Liver Cancer 1 (DLC1), a Rho GTPase with roles in proliferation, migration and invasion often mutated or deleted in various cancers [69]. None of the six samples had a mutation in RAD51 (**Table 1**), suggesting that co-mutations in RAD52 and BRCA2-BRCA1-PALB2 make RAD51 indispensable.

We also identified seven samples with various truncations of the RAD52 C-terminus (V105Wfs*7, T318Rfs*5, E320*, W386*, Y415*) (**Table 1**). All seven samples were associated with BRCA1 or BRCA2 mutations with a significant VEST or CHASM p-value. An additional sample (G399*) which also had a second mutation (M78I) did not show any significant mutations in BRCA2, BRCA1 or PALB2. The E320* truncation was identified in three different patients, and it was associated with a significant BRCA1 and BRCA2 mutation burden. Previously a RAD52 S346* truncation was associated with reduced breast cancer risk in carriers of germline BRCA2 mutations [22]. Subsequent experiments have shown that the S346* truncation affects RAD52 oligomerization and HR repair when expressed in budding yeast [70]. This truncation also shows decreased nuclear localization because it loses the C-terminus domain where the NLS sequences are (**Fig 3**) [22]. In human cells but not budding yeast S346* also shows SSA defects [22, 70]. The authors suggest that in the absence of BRCA2 function, repair relies on RAD52 which promotes mutagenic SSA. A reduction in SSA by the S346* truncation suppresses BRCA2 mutants. We also speculate that the truncations that we identified function by similar mechanisms. Remarkably, all the truncations were identified in other than breast cancers suggesting that the decrease in RAD52 activity in the BRCA2 and BRCA1 background mutations functions in a similar fashion in other cancer types.

**Fig 4:**
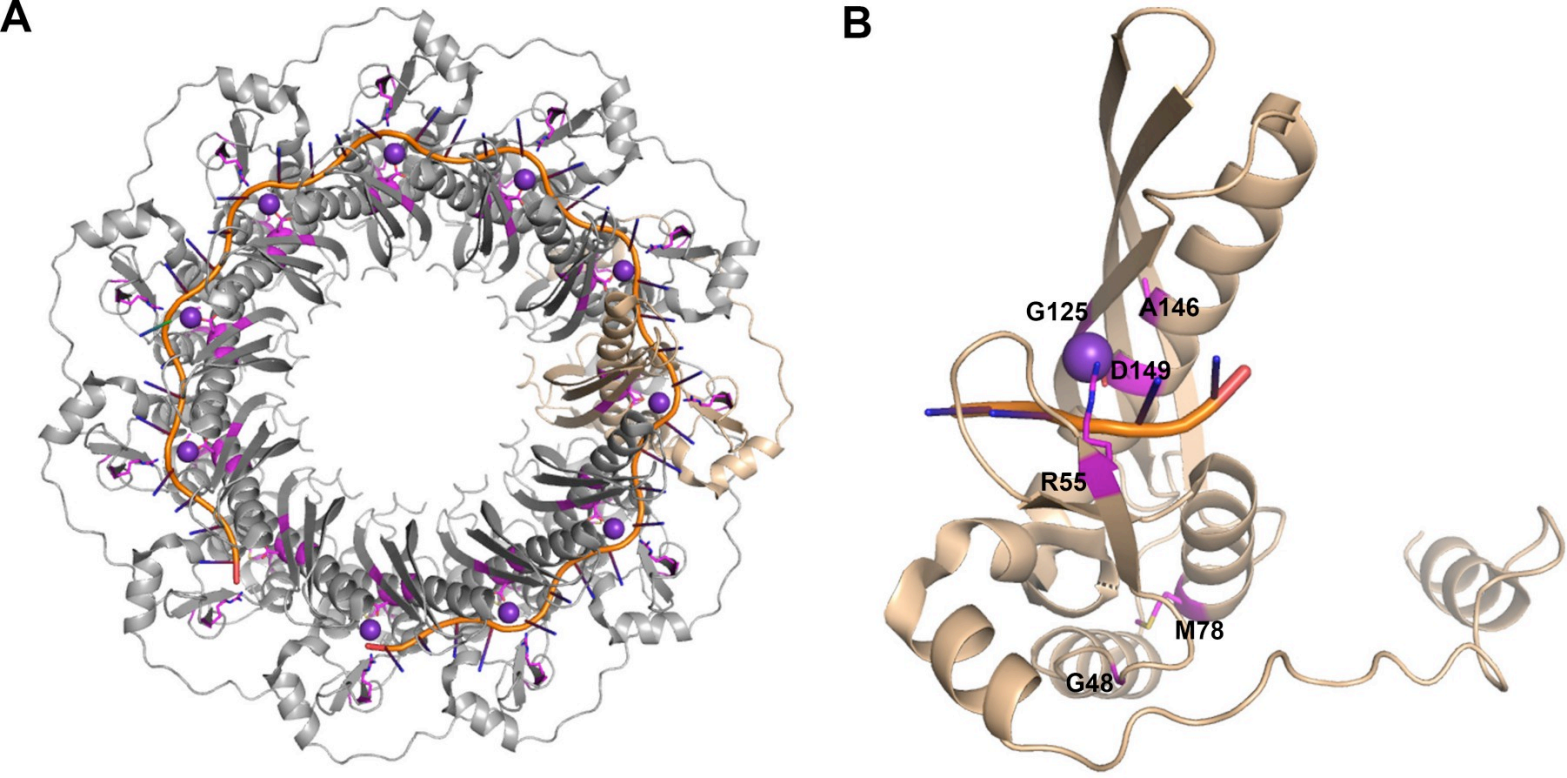
Location of mutated residues on the structure of RAD52. **A**. The structure of RAD52 with single-stranded DNA bound to the inner site (PDB ID: 5XRZ) was used to map the mutated residues, shown in magenta. The DNA is shown in orange with bases in dark blue and potassium ions are shown as purple spheres. RAD52 is shown in gray with a single monomer represented in tan. **B**. The tan single monomer is shown individually, and side chains of mutated residues shown as magenta sticks.

**Fig 5:**
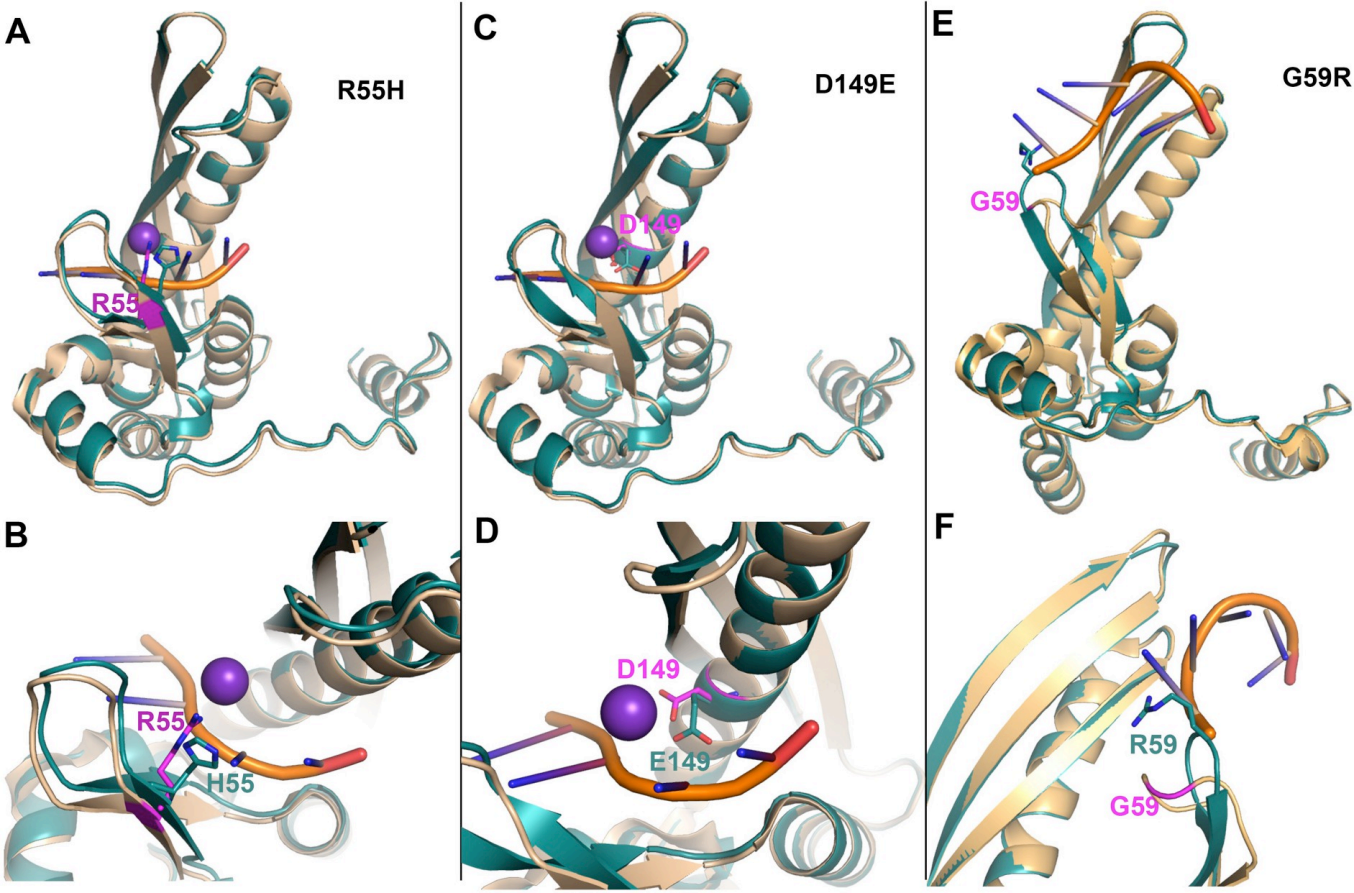
The effect of R55H, D149E, and G59R on the structure of RAD52. Structures of point mutations (cyan) were generated using homology modeling and then aligned to known structures of RAD52 (tan). The side chain of the mutated residue is shown in sticks. **A**. R55H mutant is aligned to a monomer with DNA bound to the inner site (PDB ID: 5XRZ). **B.** Zoomed in view of the R55H mutation compared to wildtype. **C**. D149E mutant is aligned to a monomer with DNA bound to the inner site (PDB ID: 5XRZ). **D.** Zoomed in view of the D149E mutation compared to wildtype. **E**. G59R mutant is aligned to a monomer with DNA bound to the outer site (PDB ID: 5XS0). **F.** Zoomed in view of the G59R mutation compared to wildtype.

*3D structure of point mutants*. To understand whether the pathogenic or driver mutations identified in the N-terminus of RAD52 (**Fig 3**, **Table 1**) affect interaction with DNA, we generated protein models (**Figs 4 and 5, S2 Fig**). Mutated residues in the N-terminal domain (**Fig 3**) were first mapped onto known crystal structures of RAD52 with DNA bound in the inner site (PDB ID: 5XRZ) and outer site (PDB ID: 5XS0) to determine if these residues were in contact with DNA or in the protein-protein interfaces of the oligomeric structure (**Fig 4A**) [71]. There are no full-length structures of RAD52 available since the C-terminal domain is flexible and interferes with crystallization. The R55 and D149 residues were observed to coordinate potassium ions that are critical for the binding of single-stranded DNA (ssDNA) in the inner site of RAD52 (**Fig 4B**). G48, M78, and A146 were not in contact with DNA, the potassium ions, or were located along the oligomerization interface. G125 is found along the oligomerization interface, however the residue is oriented so that any side chain would not point outwards. Structures of single point mutants were then generated by making the corresponding change in the N-terminal sequence of RAD52 and then using homology modeling to build the structure of each mutant sequence using SWISS-MODEL [49]. Each mutant was aligned to known crystal structures of RAD52 to then determine how the mutation would impact protein structure, DNA binding, or oligomerization. The R55H mutation would be expected to negatively impact ssDNA binding within the inner site, as it helps anchor the phosphate backbone of DNA into the binding site and is near the potassium ion (**Fig 5A and 5B**). Modeling the D149E mutation shows that it could affect the coordination of the potassium ion as the longer side chain cannot be appropriately accommodated (**Fig 5C and 5D**). The other mutants (G48D, M78I, G125C, and A146V) did not show any significant impact on the protein structure (**S2 Fig**). A RAD52 G59R mutation identified in Black women with breast cancer was shown to have decreased association with DNA [70]. We also modeled the G59R mutation [70] to see if this result could be explained structurally. The model of this mutant displayed a more extended loop structure that sterically clashes with the DNA bound to the outer site (**Fig 5E and 5F**). In addition, the arginine side chain extends out towards the DNA, which could also prevent DNA binding. This analysis indicates the R55H and to some extent the D149E mutation is likely to decrease the association of RAD52 with DNA in a manner previously shown for the G49R mutation.

## Conclusion

Because RAD52 and BRCA2-BRCA1-PALB2 represent two mechanisms by which RAD51 maybe loaded onto the resected single-stranded DNA to facilitate HR repair, co-knockdown of RAD52 and any gene from the BRCA2-BRCA1-PALB2 axis is lethal [29, 34]. Here we show that RAD52 and BRCA2-BRCA1-PALB2 co-mutations do occur in cancer cells but always in a heterozygous state. This supports the above observation that co-inactivation of these two pathways will kill the cell. No RAD52 mutation was identified that appears to significantly affect the function of its N-terminus in line with the observation that this RAD52 region is essential in the absence of BRCA2-BRCA1-PALB2 function [33].

We find that certain co-occurring RAD52 pathogenic mutations are likely to decrease the association of the protein with DNA or its oligomerization properties. Previous observations have shown that similar RAD52 mutations have a protective effect in Black women with germline BRCA2 mutations. These mutations affect the SSA, or other HR pathways facilitated by RAD52. Because RAD52 mediated repair is more mutagenic than BRCA2-BRCA1-PALB2, a downregulation of RAD52 activity is protective in the background of the BRCA2-BRCA1-PALB2 mutations. Remarkably, The R55H mutation and several C-terminal truncations are identified in metastatic or advanced stage cancers (**S1D Table**). We suspect that these co-mutations have the same effect in cancer cells, namely that they retain HR activity but with downregulated SSA which may prevent over-accumulation of damage. This may promote the viability or proliferation ability of cancer cells.

Traditionally, the yeast model system has been fundamental in unravelling mechanisms and pathways of DNA damage repair [72, 73]. Although neither BRCA1 or BRCA2 are found in yeast, a yeast assay to characterize certain ectopically expressed human BRCA mutations has also been developed [74]. This assay was used to show that certain potentially pathogenic BRCA1 mutations adversely affect HR. Interesting, the deletion of yeast RAD52 suppressed the adverse effects of these pathogenic mutations [75]. Other interesting genetic interactions between human BRCA1/2, RAD52 and other repair genes have also been uncovered in the yeast system [70, 76–79]. Thus, mutations of clinical relevance could indeed be evaluated in the yeast model and predictions about their pathogenetic can be made.

## Supporting information

S1 Fig Graphical distribution of mutations from Table 1. All mutations were graphed using the lollipop software. Please see materials and methods.
(TIF)

S2 Fig Models of G48D, M78I, G125C, and A146V RAD52 point mutants. Structures of point mutations (cyan) were generated using homology modeling and then aligned to known structures of RAD52 (tan). The side chain of the mutated residue is shown in sticks. Zoomed in view of **A.** G48D, **B.** M78I, **C.** G125C, and **D.** A146V mutants compared to wildtype, which are aligned to a monomer with DNA bound to the inner site (PDB ID: 5XRZ).
(TIF)

S1 Table BRCA2, BRCA1 and PALB2 co-occurring mutations with RAD52. All co-occurring mutations reported on COSMIC regardless of their pathogenicity or driver status.
(DOCX)

S2 Table Co-occurrence or mutual exclusivity statistical values by cancer type. cBioPortal statistics on co-occurring mutations. The data in this table was used for Fig 2C & 2D.
(DOCX)

S3 Table Pathogenicity and driver probability values using the OPEN Cravat tool. **A.** VEST and CHASM p-values for co-occurring RAD52 mutations listed in Table 1. For easy identification each mutation is highlighted by a different color. Those with significant p-values are indicated with one star (either VEST or CHASM) or two stars (both). **B**. VEST and CHASM p-values for co-occurring BRCA2 mutations listed in Table 1. For easy identification each mutation is highlighted by a different color. Those with significant p-values are indicated with one star (either VEST or CHASM) or two stars (both). **C**. VEST and CHASM p-values for co-occurring BRCA1 mutations listed in Table 1. For easy identification each mutation is highlighted by a different color. Those with significant p-values are indicated with one star (either VEST or CHASM) or two stars (both). **D**. VEST and CHASM p-values for co-occurring PALB2 mutations listed in Table 1. For easy identification each mutation is highlighted by a different color. Those with significant p-values are indicated with one star (either VEST or CHASM) or two stars (both). **E**. Sample information for the identified mutations. **F**. Coordinates for lollipop figures in S1 Fig.
(XLSX)
