# Supplementary figures and images for: Pan-cancer analysis of co-occurring mutations in RAD52 and the BRCA1-BRCA2-PALB2 axis in human cancers
Source: PLoS One. 2022 Sep 15;17(9):e0273736. doi: 10.1371/journal.pone.0273736 (PMC9477347; doi:10.1371/journal.pone.0273736)

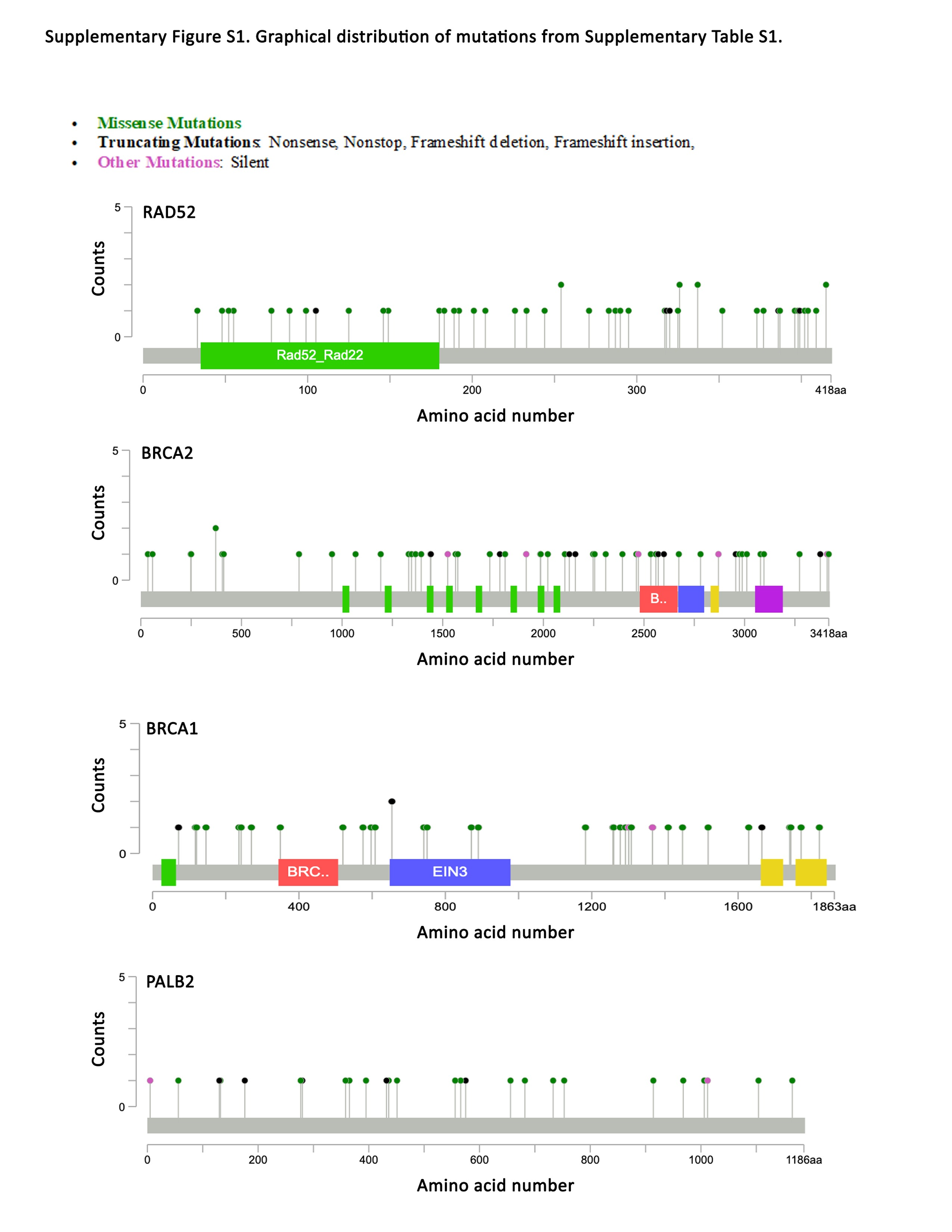

Supplement: S1 Fig — All mutations were graphed using the lollipop software. Please see materials and methods. (TIF) [file pone.0273736.s001.tif]

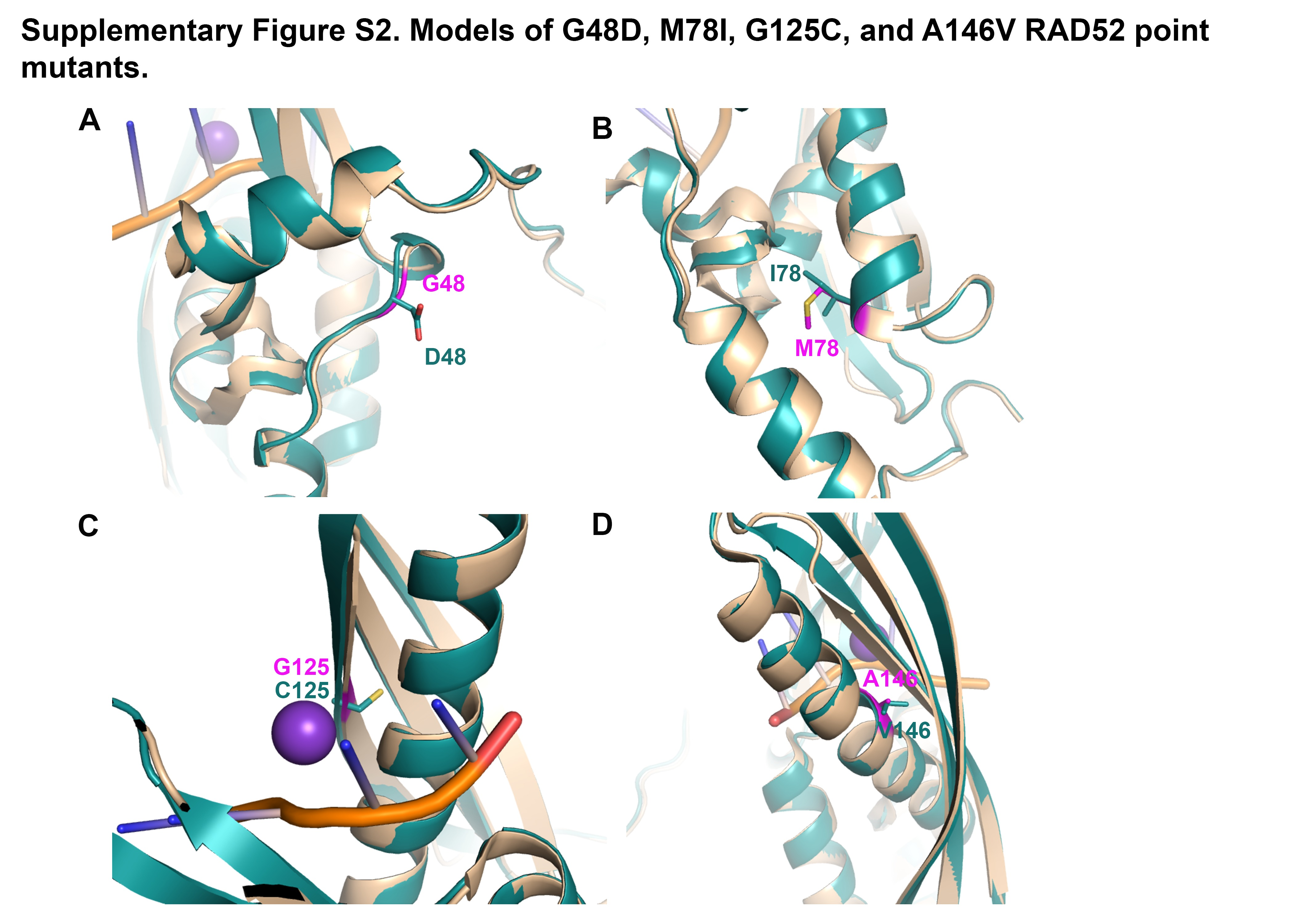

Supplement: S2 Fig — Structures of point mutations (cyan) were generated using homology modeling and then aligned to known structures of RAD52 (tan). The side chain of the mutated residue is shown in sticks. Zoomed in view of A. G48D, B. M78I, C. G125C, and D. A146V mutants compared to wildtype, which are aligned to a monomer with DNA bound to the inner site (PDB ID: 5XRZ). (TIF) [file pone.0273736.s002.tif]
