# Supplementary material for: Pan-cancer analysis of co-occurring mutations in RAD52 and the BRCA1-BRCA2-PALB2 axis in human cancers
Source: PLoS One. 2022 Sep 15;17(9):e0273736. doi: 10.1371/journal.pone.0273736 (PMC9477347; doi:10.1371/journal.pone.0273736)
Supplement: S1 Table — All co-occurring mutations reported on COSMIC regardless of their pathogenicity or driver status. (DOCX) [file pone.0273736.s003.docx]

**Supplementary Table S1. BRCA2, BRCA1 and PALB2 co-occurring mutations with RAD52.**

| **^1^Sample** | **RAD52** | **BRCA2** | **BRCA1** | **PALB2** | **Tissue (histology)** |
| --- | --- | --- | --- | --- | --- |
| TCGA-AA-3510-01 | E320* | E2599* | E1258D | L451V | Large intestine (adenocarcinoma) |
| P-0001808-T01-IM3 | E320* | E2129*  K2673T | K250N | None | Large intestine (adenocarcinoma) |
| P-0005824-T01-IM5 | E320* | S1331Y, D1345Y  T2255A, L3011I | E1665*, E275K, K127Q, L1260V, S741Y | K436N, R753Q, V132A | Large intestine (adenocarcinoma) |
| T3704 | D409V | E2571*, T1915= | K654Sfs*47 | None | Large intestine (adenocarcinoma) |
| P-0006960-T01-IM5 | S373N | D1575Y, S2247I, A1439T, A1564T, S2462Y, A248T | L750I | W575*  S395Y | Large intestine (adenocarcinoma) |
| P-0006612-T01-IM5 | R180C | S2559Y, F1192C, E1734K, S786Y | S1292Y | D56Y  P358S | Large intestine (adenocarcinoma) |
| P-0005230-T01-IM5 | T254M | V3079I | S1448N | None | Large intestine (adenocarcinoma) |
| P-0002671-T01-IM3 | L244M | M2393T | None | H130Tfs*47 | Large intestine (adenocarcinoma) |
| P-0000682-T01-IM3 | D337N | T1067S | None | None | Large intestine (adenocarcinoma) |
| 526LT | R55H | N372H | K1183R, P871L | None | Large intestine (adenocarcinoma) |
| B1-CaA | S226F | E3096K, D3410= | E349V | R566H | Large intestine (adenocarcinoma) |
| P-0008729-T01-IM5 | E402K | None | G890W | None | Large intestine (adenocarcinoma) |
| P-0010783-T01-IM5 | I233M | None | K654Sfs*47 | None | Large intestine (adenocarcinoma) |
| P-0013492-T01-IM5 | G48D | None | None | H432Ffs*9 | Large intestine (adenocarcinoma) |
| TCGA-D3-A2JP-06 | K192N | L35F | Q1409H | None | Skin (malignant melanoma) |
| WD_06 | D149E | T2310I | None | None | Skin (squamous cell carcinoma) |
| TCGA-ER-A19P-06 | G125C | Q1987E | None | None | Skin (malignant melanoma) |
| P-0010649-T01-IM5 | C208R | A2534V | None | P656L | Skin (squamous cell carcinoma) |
| PT37 | T189I | None | S146F | None | Skin (basal cell carcinoma) |
| P-0001042-T01-IM3 | P183H, R396H | None | T236I | D277N | Skin (squamous cell carcinoma) |
| PD42111a | L325F | S1985F, V250M | None | R365K | Skin (malignant melanoma) |
| TCGA-B5-A1MX-01 | G399*, M78I | F3273L, K2472=, Q2870= | None | None | Endometrium (endometrioid carcinoma) |
| TCGA-DI-A0WH-01 | V105Wfs*7 | N2781D | S1301= | None | Endometrium (endometrioid carcinoma) |
| TCGA-AX-A2HD-01 | A146V | E1441*, G405R | None |  | Endometrium (endometrioid carcinoma) |
| P-0005285-T01-IM5 | T89M | N1784Tfs*7 | None | N280Tfs*8 | Endometrium (endometrioid carcinoma) |
| TCGA-VQ-A91D-01 | T398A | A1393V | G1366= | S682P | Stomach (adenocarcinoma) |
| 155 | E201V | None | D120G | None | Stomach (carcinoma) |
| TCGA-CG-5721-01 | V317A  R180= | S2022L, V1810I, H1525= | None | T733A,  T1012= | Stomach (adenocarcinoma) |
| TCGA-CG-5723-01 | S287G | I3418S | None | S1165L | Stomach (adenocarcinoma) |
| P-0001774-T01-IM3 | A352T | E58K, I2974T | None | I556F | Prostate (adenocarcinoma) |
| SC_9047 | R271Q | None | H270R | None | Prostate (carcinoma) |
| TCGA-44-3919-01 | H404D | S3376* | None | None | Lung (adenocarcinoma) |
| P-0000671-T01-IM3 | I52L | P412L | A1308S | None | Lung (adenocarcinoma) |
| LUAD-CHTN-MAD06-00668 | Q377L | None | None | G1104A,  P5= | Lung (adenocarcinoma) |
| CHG-14-15016T | Y415C | E2956*, V950F, C1365F | None | None | Liver (neoplasm) |
| CHG-13-09089T | A33E | S2988I | H117N | F914L | Liver (neoplasm) |
| TCGA-BC-A112-01 | E326Q | None | A1629T | None | Liver (hepatocellular carcinoma) |
| P-0006124-T01-IM5 | S283L | Q2159* | S1518P, E597K | L176Nfs*3 | Urinary tract (transitional cell carcinoma) |
| P-0011440-T01-IM5 | T318Rfs*5 | None | I1744V | None | Urinary tract (small cell carcinoma) |
| RMS112 | Y415* | N372H  P2107T | None | None | Soft tissue (rhabdomyosarcoma) |
| P-0000947-T01-IM3 | P295S | None | K608N | None | Breast (ER-PR positive carcinoma) |
| P-0008994-T01-IM5 | N99D | None | R71Kfs*10 | None | Breast (lobular carcinoma) |
| MBC556 | D337E | None | None | L1006F | Breast (ductal carcinoma) |
| P-0006571-T01-IM5 | T254R | None | T1741I | None | Salivary gland (squamous cell carcinoma) |
| P-0008388-T01-IM5 | D387N | None | S242N | None | CNS (oligodendroglioma grade III) |
| P-0004978-T01-IM5 | A290P | None | None | A968V | Testis (seminoma) |
| Pat_44_B | W386* | None | R1772Q, G1822D | None | Not specified |

^1^All samples analyzed are from COSMIC which deposits data from TCGA as well as other studies. TCGA samples labels start with “TCGA” (e.g., TCGA-AA-3510-01), while non-TCGA samples have different labels. Further information for each sample in this table is given in “Supplementary Table S3E” including a PubMed ID (if available) for the manuscript where the mutation was first described.
