# Supplementary material for: Pan-cancer analysis of co-occurring mutations in RAD52 and the BRCA1-BRCA2-PALB2 axis in human cancers
Source: PLoS One. 2022 Sep 15;17(9):e0273736. doi: 10.1371/journal.pone.0273736 (PMC9477347; doi:10.1371/journal.pone.0273736)
Supplement: S2 Table — cBioPortal statistics on co-occurring mutations. The data in this table was used for Fig 2C & 2D. (DOCX) [file pone.0273736.s004.docx]

**Supplementary Table S2. Co-occurrence or mutual exclusivity statistical values by cancer type.** Please see “Materials and Methods” for interpretation of this table.

| Tissue | Gene A | Gene B | Neither | A Not B | B Not A | Both | Log2 Odds Ratio | p-Value | q-Value | Tendency |
| --- | --- | --- | --- | --- | --- | --- | --- | --- | --- | --- |
| Adrenal gland | RAD52 | BRCA2 | 895 | 6 | 61 | 1 | 1.290 | 0.373 | 0.747 | Co-occurrence |
|  | RAD52 | BRCA1 | 928 | 7 | 28 | 0 | <-3 | 0.813 | 0.909 | Mutual exclusivity |
|  | RAD52 | PALB2 | 943 | 7 | 13 | 0 | <-3 | 0.909 | 0.909 | Mutual exclusivity |
| Ampulla of Vater | RAD52 | BRCA2 | 152 | 0 | 8 | 0 | >3 | 1.000 | 1.000 | Co-occurrence |
|  | RAD52 | BRCA1 | 155 | 0 | 5 | 0 | >3 | 1.000 | 1.000 | Co-occurrence |
|  | RAD52 | PALB2 | 158 | 0 | 2 | 0 | >3 | 1.000 | 1.000 | Co-occurrence |
| Biliary tract | RAD52 | BRCA2 | 638 | 1 | 20 | 0 | <-3 | 0.970 | 0.988 | Mutual exclusivity |
|  | RAD52 | BRCA1 | 649 | 1 | 9 | 0 | <-3 | 0.986 | 0.988 | Mutual exclusivity |
|  | RAD52 | PALB2 | 650 | 1 | 8 | 0 | <-3 | 0.988 | 0.988 | Mutual exclusivity |
| Bladder/Urinary tract | RAD52 | BRCA2 | 743 | 12 | 65 | 2 | 0.930 | 0.318 | 0.382 | Co-occurrence |
|  | RAD52 | BRCA1 | 772 | 12 | 36 | 2 | 1.838 | 0.134 | 0.284 | Co-occurrence |
|  | RAD52 | PALB2 | 774 | 13 | 34 | 1 | 0.808 | 0.459 | 0.459 | Co-occurrence |
| Bone | RAD52 | BRCA2 | 217 | 1 | 1 | 0 | <-3 | 0.995 | 0.995 | Mutual exclusivity |
|  | RAD52 | BRCA1 | 217 | 1 | 1 | 0 | <-3 | 0.995 | 0.995 | Mutual exclusivity |
|  | RAD52 | PALB2 | 217 | 1 | 1 | 0 | <-3 | 0.995 | 0.995 | Mutual exclusivity |
| Bowel | RAD52 | BRCA2 | 1278 | 25 | 114 | 4 | 0.843 | 0.216 | 0.259 | Co-occurrence |
|  | RAD52 | BRCA1 | 1340 | 25 | 52 | 4 | 2.044 | 0.025 | 0.037 | Co-occurrence |
|  | RAD52 | PALB2 | 1354 | 28 | 38 | 1 | 0.348 | 0.557 | 0.557 | Co-occurrence |
| Breast | RAD52 | BRCA2 | 2047 | 43 | 96 | 4 | 0.988 | 0.164 | 0.341 | Co-occurrence |
|  | RAD52 | BRCA1 | 2053 | 44 | 90 | 3 | 0.637 | 0.322 | 0.483 | Co-occurrence |
|  | RAD52 | PALB2 | 2063 | 46 | 80 | 1 | -0.835 | 0.475 | 0.546 | Mutual exclusivity |
| CNS/Brain | RAD52 | BRCA2 | 2384 | 39 | 42 | 6 | >3 | <0.001 | <0.001 | Co-occurrence |
|  | RAD52 | BRCA1 | 2401 | 38 | 25 | 7 | >3 | <0.001 | <0.001 | Co-occurrence |
|  | RAD52 | PALB2 | 2412 | 41 | 14 | 4 | >3 | <0.001 | <0.001 | Co-occurrence |
| Cervix | RAD52 | BRCA2 | 271 | 2 | 14 | 0 | <-3 | 0.905 | 0.965 | Mutual exclusivity |
|  | RAD52 | BRCA1 | 270 | 2 | 15 | 0 | <-3 | 0.898 | 0.965 | Mutual exclusivity |
|  | RAD52 | PALB2 | 280 | 2 | 5 | 0 | <-3 | 0.965 | 0.965 | Mutual exclusivity |
| Esophagus/Stomach | RAD52 | BRCA2 | 1104 | 22 | 95 | 10 | 2.401 | <0.001 | <0.001 | Co-occurrence |
|  | RAD52 | BRCA1 | 1160 | 29 | 39 | 3 | 1.621 | 0.092 | 0.092 | Co-occurrence |
|  | RAD52 | PALB2 | 1181 | 24 | 18 | 8 | >3 | <0.001 | <0.001 | Co-occurrence |
| Eye | RAD52 | BRCA2 | 105 | 0 | 1 | 0 | >3 | 1.000 | 1.000 | Co-occurrence |
|  | RAD52 | BRCA1 | 105 | 0 | 1 | 0 | >3 | 1.000 | 1.000 | Co-occurrence |
|  | RAD52 | PALB2 | 106 | 0 | 0 | 0 | >3 | 1.000 | 1.000 | Co-occurrence |
| Head and Neck | RAD52 | BRCA2 | 840 | 17 | 25 | 3 | 2.568 | 0.023 | 0.135 | Co-occurrence |
|  | RAD52 | BRCA1 | 848 | 20 | 17 | 0 | <-3 | 0.676 | 0.748 | Mutual exclusivity |
|  | RAD52 | PALB2 | 857 | 19 | 8 | 1 | 2.495 | 0.187 | 0.373 | Co-occurrence |
| Kidney | RAD52 | BRCA2 | 1417 | 9 | 19 | 0 | <-3 | 0.887 | 0.945 | Mutual exclusivity |
|  | RAD52 | BRCA1 | 1424 | 9 | 12 | 0 | <-3 | 0.928 | 0.945 | Mutual exclusivity |
|  | RAD52 | PALB2 | 1427 | 9 | 9 | 0 | <-3 | 0.945 | 0.945 | Mutual exclusivity |
| Liver | RAD52 | BRCA2 | 1076 | 2 | 22 | 0 | <-3 | 0.960 | 0.995 | Mutual exclusivity |
|  | RAD52 | BRCA1 | 1083 | 2 | 15 | 0 | <-3 | 0.973 | 0.995 | Mutual exclusivity |
|  | RAD52 | PALB2 | 1095 | 2 | 3 | 0 | <-3 | 0.995 | 0.995 | Mutual exclusivity |
| Lung | RAD52 | BRCA2 | 2505 | 46 | 112 | 2 | -0.040 | 0.662 | 0.662 | Mutual exclusivity |
|  | RAD52 | BRCA1 | 2533 | 47 | 84 | 1 | -0.640 | 0.543 | 0.662 | Mutual exclusivity |
|  | RAD52 | PALB2 | 2568 | 47 | 49 | 1 | 0.157 | 0.600 | 0.662 | Co-occurrence |
| Lymphoid | RAD52 | BRCA2 | 2990 | 7 | 10 | 0 | <-3 | 0.977 | 0.991 | Mutual exclusivity |
|  | RAD52 | BRCA1 | 2984 | 7 | 16 | 0 | <-3 | 0.963 | 0.991 | Mutual exclusivity |
|  | RAD52 | PALB2 | 2996 | 7 | 4 | 0 | <-3 | 0.991 | 0.991 | Mutual exclusivity |
| Myeloid | RAD52 | BRCA2 | 1176 | 3 | 4 | 0 | <-3 | 0.990 | 0.997 | Mutual exclusivity |
|  | RAD52 | BRCA1 | 1172 | 3 | 8 | 0 | <-3 | 0.980 | 0.997 | Mutual exclusivity |
|  | RAD52 | PALB2 | 1179 | 3 | 1 | 0 | <-3 | 0.997 | 0.997 | Mutual exclusivity |
| Ovary/Fallopian tube | RAD52 | BRCA2 | 452 | 35 | 21 | 3 | 0.884 | 0.261 | 0.630 | Co-occurrence |
|  | RAD52 | BRCA1 | 453 | 34 | 20 | 4 | 1.414 | 0.094 | 0.562 | Co-occurrence |
|  | RAD52 | PALB2 | 465 | 38 | 8 | 0 | <-3 | 0.537 | 0.697 | Mutual exclusivity |
| Pancreas | RAD52 | BRCA2 | 950 | 18 | 14 | 1 | 1.915 | 0.255 | 0.391 | Co-occurrence |
|  | RAD52 | BRCA1 | 945 | 18 | 19 | 1 | 1.466 | 0.326 | 0.391 | Co-occurrence |
|  | RAD52 | PALB2 | 957 | 19 | 7 | 0 | <-3 | 0.872 | 0.872 | Mutual exclusivity |
| Peripheral nervous system | RAD52 | BRCA2 | 214 | 2 | 1 | 0 | <-3 | 0.991 | 0.995 | Mutual exclusivity |
|  | RAD52 | BRCA1 | 212 | 2 | 3 | 0 | <-3 | 0.972 | 0.995 | Mutual exclusivity |
|  | RAD52 | PALB2 | 214 | 2 | 1 | 0 | <-3 | 0.991 | 0.995 | Mutual exclusivity |
| Pleura | RAD52 | BRCA2 | 105 | 2 | 1 | 0 | <-3 | 0.981 | 0.991 | Mutual exclusivity |
|  | RAD52 | BRCA1 | 105 | 2 | 1 | 0 | <-3 | 0.981 | 0.991 | Mutual exclusivity |
|  | RAD52 | PALB2 | 105 | 2 | 1 | 0 | <-3 | 0.981 | 0.991 | Mutual exclusivity |
| Prostate | RAD52 | BRCA2 | 1715 | 26 | 98 | 5 | 1.751 | 0.026 | 0.031 | Co-occurrence |
|  | RAD52 | BRCA1 | 1779 | 25 | 34 | 6 | >3 | <0.001 | <0.001 | Co-occurrence |
|  | RAD52 | PALB2 | 1776 | 22 | 37 | 9 | >3 | <0.001 | <0.001 | Co-occurrence |
| Skin | RAD52 | BRCA2 | 1200 | 29 | 133 | 8 | 1.316 | 0.030 | 0.045 | Co-occurrence |
|  | RAD52 | BRCA1 | 1236 | 32 | 97 | 5 | 0.993 | 0.135 | 0.162 | Co-occurrence |
|  | RAD52 | PALB2 | 1270 | 34 | 63 | 3 | 0.831 | 0.262 | 0.262 | Co-occurrence |
| Soft tissue | RAD52 | BRCA2 | 560 | 7 | 18 | 3 | >3 | 0.004 | 0.024 | Co-occurrence |
|  | RAD52 | BRCA1 | 573 | 10 | 5 | 0 | <-3 | 0.918 | 0.975 | Mutual exclusivity |
|  | RAD52 | PALB2 | 575 | 10 | 3 | 0 | <-3 | 0.950 | 0.975 | Mutual exclusivity |
| Testis | RAD52 | BRCA2 | 141 | 8 | 0 | 0 | >3 | 1.000 | 1.000 | Co-occurrence |
|  | RAD52 | BRCA1 | 141 | 8 | 0 | 0 | >3 | 1.000 | 1.000 | Co-occurrence |
|  | RAD52 | PALB2 | 141 | 8 | 0 | 0 | >3 | 1.000 | 1.000 | Co-occurrence |
| Thymus | RAD52 | BRCA2 | 152 | 1 | 2 | 0 | <-3 | 0.987 | 1.000 | Mutual exclusivity |
|  | RAD52 | BRCA1 | 153 | 1 | 1 | 0 | <-3 | 0.994 | 1.000 | Mutual exclusivity |
|  | RAD52 | PALB2 | 154 | 1 | 0 | 0 | >3 | 1.000 | 1.000 | Co-occurrence |
| Thyroid | RAD52 | BRCA2 | 484 | 1 | 2 | 0 | <-3 | 0.996 | 1.000 | Mutual exclusivity |
|  | RAD52 | BRCA1 | 484 | 1 | 2 | 0 | <-3 | 0.996 | 1.000 | Mutual exclusivity |
|  | RAD52 | PALB2 | 486 | 1 | 0 | 0 | >3 | 1.000 | 1.000 | Co-occurrence |
| Uterus | RAD52 | BRCA2 | 575 | 19 | 86 | 6 | 1.078 | 0.104 | 0.104 | Co-occurrence |
|  | RAD52 | BRCA1 | 608 | 20 | 53 | 5 | 1.520 | 0.052 | 0.062 | Co-occurrence |
|  | RAD52 | PALB2 | 626 | 21 | 35 | 4 | 1.768 | 0.047 | 0.062 | Co-occurrence |
| Vulva/Vagina | RAD52 | BRCA2 | 12 | 1 | 2 | 0 | <-3 | 0.867 | 1.000 | Mutual exclusivity |
|  | RAD52 | BRCA1 | 14 | 1 | 0 | 0 | >3 | 1.000 | 1.000 | Co-occurrence |
|  | RAD52 | PALB2 | 14 | 1 | 0 | 0 | >3 | 1.000 | 1.000 | Co-occurrence |
| Other (mixed cancer types) | RAD52 | BRCA2 | 24311 | 437 | 1083 | 77 | 1.984 | <0.001 | <0.001 | Co-occurrence |
|  | RAD52 | BRCA1 | 24766 | 454 | 628 | 60 | 2.382 | <0.001 | <0.001 | Co-occurrence |
|  | RAD52 | PALB2 | 24990 | 487 | 404 | 27 | 1.778 | <0.001 | <0.001 | Co-occurrence |
